# EPIDEMIOLOGY AND TREATMENT OF MONTEGGIA LESION IN ADULTS: SERIES OF 44 CASES

**DOI:** 10.1590/1413-785220162401152249

**Published:** 2016

**Authors:** Roberto Suarez, Antonio Barquet, Rodrigo Fresco

**Affiliations:** 1. Asociación Española, Banco de Seguros del Estado, Montevideo, Uruguay; 2. Universidad de la Republica, Asociación Española, Banco de Seguros del Estado, Montevideo, Uruguay; 3. Universidad de la República, Montevideo, Uruguay

**Keywords:** Monteggia's fracture, Wounds and Injuries, Surgical Procedures, Operative

## Abstract

**Objective:**

To analyze the epidemiology, treatment and outcome of a series of adult patients with Monteggia lesion treated in Uruguayan institutions.

**Methods:**

This is a retrospective article, we retrospectively identified from two Uruguayan institutions 44 adult patients with Monteggia lesion and analyzed their characteristics including Bado classification, associated injuries, treatment modality and outcome (Morrey score).

**Results:**

Using Bado classification, 23 cases (52%) were type II, 12 (27%) type I, seven (16%) type IV and two cases (5%) type III. Associated lesions were radial head fractures, found in 15 patients, coronoid ipsilateral fractures in seven patients, and neurological injuries in four. Radial head dislocation was reduced in 93% of the cases with closed maneuvers. Ulna fractures underwent open reduction and internal fixation in all 30 cases using 3.5 mm DCP plates. Complications after surgery occurred in 21 cases. Revision surgery was done in 15 cases. Outcomes after primary and revision surgery were good or excellent in 37 cases.

**Conclusions:**

In our series we observed that Monteggia lesion in adults is a serious injury with a high number of complications that often require revision surgeries. ***Level of Evidence IV, Retrospective Study, Case Series.***

## INTRODUCTION

Monteggia lesion, defined as an associated fracture at any segment of the ulna associated to a radial head dislocation is a recognized serious injury, however rare. It represents 0.7% of all elbow fractures and dislocations in adult patients.^1^ ^-^ ^6^

Bado, in 1956, classified this injury as follows: type I, anterior dislocation and anterior angulation; type II, posterior dislocation and posterior angulation; type III, lateral dislocation and lateral angulation; and type IV, proximal third fracture of both bones and anterior dislocation of the radial head.1,5 Type III, usually found only in children, is an anterior or anterolateral dislocation of the radial head combined with an ulna metaphysis fracture with lateral angulation (usually in greenstick). Type IV is extremely uncommon.^7^ It is an ulna fracture of the middle or proximal third, with an anterior luxation of the radial head and an upper radial third fracture which is distal to the bicipital tuberosity. It can be considered as a variant of type I, showing in addition a radial proximal third fracture.

Jupiter subclassified Type II lesions as follows: IIa, ulna fracture involving the distal end of the olecranon and the coronoid process; IIb, metaphyseal-diaphyseal fracture, distal to the coronoid process; llc, diaphyseal fracture of the ulna; and IId, fracture of the ulna halfway through the bone.^8^

The diagnosis of the elbow component is frequently missed initially and the treatment may be difficult, with relatively high unsatisfactory outcomes. There are the so called equivalents, which result of a common pathogenic mechanism, as described by Bado, but do not correspond to the typical Monteggia lesion.^1^

A consensus exists that treatment must be surgical and that this lesion needs to be treated urgently to improve outcomes.^9^ ^-^ ^11^

Due to its low frequency, the number of series with a significant number of patients is small^3^ ^,^ ^4^ and we have not identified any series published from Latin American countries. With the objective of further describing this rare injury, we retrospectively identified patients with Monteggia lesion from two Uruguayan institutions and described their characteristics, treatment and outcome.

## MATERIAL AND METHODS

The clinical records and radiographs of all adult patients (>18 years old) with Monteggia lesion assisted at two institutions in Montevideo, Uruguay between 2006 and 2012 were retrospectively reviewed.

This research waived the requirement of approval of the Ethics Committee and Informed Consent form because this was a retrospective study (review of medical records)

The following parameters were analyzed: gender, age, mechanism of injury, type of lesion according to Bado classification and Jupiter subclassification, type of lesion (closed or open), associated fractures of the radial head and coronoid process according to Mason and Morrey classifications, respectively.^12^ ^,^ ^13^

Associated neurological or vascular lesions, treatment, complications and outcomes (using Morrey's scale for evaluation of the functional outcome)^14^ were analyzed. (Annex 1)

Statistical analysis of differences was performed using the two-tailed Fisher's exact test, considering as significant *p* values lower than 0.05.

## RESULTS

We identified 44 patients with a minimum follow-up after surgery of 18 months. The patients' main characteristics are reported in Table 1, while data on treatment, complications and outcomes are shown in Table 2.

**Table 1:** Main characteristics of the 44 patients included.

Absolute frequency	%
n	44	100
Gender
Men	27	61
Women	17	39
Bado classification
Type I	17	38
Type II	23	52
Type III	2	5
Type IV	2	5
Jupiter classification (of Bado type II; n=23)
2a	7	31
2b	12	52
2c	1	4
2d	3	13
At least 1 associated injury (n: 15)	15	34
	Bado Type I: 2	
	Bado Type II: 13	
Radial head fractures	15	34
	Bado Type I: 2/17	
	Bado Type II: 13/23	
Coronoid fracture	7 (all Bado Type II)	16
Open fracture	8	16
	Bado Type I: 6	
	Bado Type II: 2	
Ipsilateral injuries of the upper limb	9	20
	Bado Type I: 7	
	Bado Type II: 2	
Vascular lesions	0	0
Neurological lesions	4 (all Bado Type I)	10

**Table 2:** Treatment, complications and outcomes.

Absolute Frequency	%
Ulna osteosynthesis	44	100
DCP 3.5 mm	30	68
3.5 mm Reconstruction plate	1	2
1/3 tube	1	2
Cerclage	8	18
External fixation + DCP	4	9
Complications	21	48
Reoperations	15	34
Morrey´s score
Good and excellent	37	84
Bad	7	16

Seventeen patients presented type I lesion (38%), 23 presented type II (52%), two presented type III (5%), and two presented type IV (5%). Regarding the mechanism of Monteggia lesion, in 14 cases (32%) it was caused by high-energy fracture in young patients (< 45 years old), being all Bado I lesions. In 30 cases (68%) Monteggia lesions were due to a low-energy trauma in older patients, being three type I lesions and the remaining, type II lesions.

According to the Jupiter's subclassification of Bado type II lesions, seven cases were type IIa (30%), 12 cases were type IIb (52%), one was type IIc (4%) and three were type IId (13%).

Patients with Bado type I lesion had median age of 32 years old and were predominantly men (75% vs 25%), while patients with type II had a median age of 49 years old and the majority were women (57% *vs* 43%).

Regarding the associated fractures, radial head fractures were observed in two patients (11%) with type I lesions, and in 13 patients (56%) with type II lesions. Radial head fractures have been classified according to Mason's classification, eight were type 3 (55%), six were type II (40%) and one type I (5%).

In type I fractures there were no coronoid fractures, whereas in type II fractures, they were found in seven cases (30%). Of all coronoid fractures, seven cases were type III according to Morrey's classification (85%), one case was type II (15%) and there were no type I cases. All coronoid fractures were associated with radial head fractures.

Regarding the association with neurological injuries, these were observed in four patients with type I lesion (23%), and none in patients with type II lesion. No vascular lesions were observed whatsoever.

The association of Monteggia lesions with other ipsilateral fractures of the upper limb was observed in seven patients with Bado type I (58%) and in two patients with Bado type II (8%). 

For the fixation of the ulnar fracture, osteosynthesis with a 3.5 mm DCP plate was performed as single implant in 30 cases (68%), with one-third tubular plate in two cases (4%), with 3.5 mm DCP plate and external fixators in four cases (8%), with 3.5 mm reconstruction plate in one case (3%) and cerclage in eight cases (18%).

In 14 cases (93%) the dislocation of the radial head was treated with a closed reduction of the ulna and in one case (7%) an open reduction was necessary.

Among the patients that presented fractures of the radial head, in two cases (12%) a non-surgical treatment was employed, in five cases (35%) open reduction and internal fixation was performed, in five cases (35%) a complete excision was performed, and in three cases (17%) a partial excision was performed. Prosthetic replacement was not used in any case.

In 21 cases (48%) complications occurred in the first surgery. The most frequent complications were: implant loosening, misalignment of the ulna, radio ulnar dislocation, nonunion of the ulna, necrosis of the radial head, postoperative infection, heterotopic ossification, loosening of osteosynthesis of radial head and neck, delayed consolidation of the neck of the radius fracture, radio-ulnar synostosis, deficiency neuropathy, and posterolateral rotatory instability. Fifteen patients (34%) that suffered complications needed reoperation.

Taking into consideration the method used for stabilization of ulna fractures, four of 31 patients that had plates (DCP and reconstruction) required reoperation (13%), while 11 out of 13 (85%) needed reoperation when a different osteosynthesis method was used.

Regarding functional results, these were satisfactory, good or excellent in 37 cases (84%), and unsatisfactory or bad in seven cases (16%). Of those seven patients with unsatisfactory or poor results, 71% had type I lesions (12/05), while 28% had type II lesions (21/02; p=0.1).

There were no significant differences in the functional results (excellent/good/ satisfactory vs. unsatisfactory/poor) in patients with neurological lesions, open fractures and ipsilateral injuries. On the other hand, there were differences in these results among patients with radial head fractures (8/29 good results vs. 5/2 bad results, p=0.0175) and with coronoid-associated fracture (3/34 good results vs. 3/4 bad results, p=0.038). In ipsilateral injuries of the upper limb, there were six good results out of 37 (31/6) and three bad results out of seven (3/4; p=0.97).

Some factors were associated with negative outcomes: radial head fractures, coronoid fractures, treatment using implants different from 3.5 mm DCP plates as well as the need of reoperations. Statistically, there were no significant results to conclude whether type I or type II influence the results. The same occurs regarding ipsilateral injuries, open fractures and neurological lesions. 

## DISCUSSION

We have reported one of the largest series of adult patients with Monteggia lesion and the first, to our knowledge, in Latin America. The other series with a similar number of patients are those from Korner et al.^2^ and Ring et al.^3^

We have shown, as these authors, a preponderance of type I lesions among young patients, resulting from high-energy trauma and associated with a higher frequency of open fractures, neurological lesions and ipsilateral injuries of the upper limb. Some authors have reported that these associated lesions are linked to a poorer prognosis.15 In our study, however, the presence of such lesions had no impact on the functional outcomes according to Morrey's score.

On the other hand, patients with type II lesions tend to be older and to have a different pattern of associated injuries such as radial head and coronoid fractures, the latter only occurring in our series in cases combined with radial head fractures. These type II lesions commonly result from less severe trauma in older patients than the ones with type I. The association with other fractures can be due to the coexistence of associated bone weakness. Moreover, the presence of associated fractures, the frequent requirement of additional surgical procedure and the typically more difficult rehabilitation process can lead to poorer functional outcomes, as seen in our series and in the studies by Korner et al.^2^ and Ring et al.^3^

Regarding the treatment of Monteggia lesions, it is essential to provide anatomic reduction and stable fixation of the ulnar fractures with 3.5 mm DCP, LC-DCP or eventually 3.5 mm reconstruction plates. As we have seen in our study, using 3.5 mm plates, both DCP and reconstruction ones, provides a more stable fixation, making the need for a second surgery less likely, therefore improving chances of better functional prognosis by avoiding reoperations.^18^ ^,^ ^19^

The anatomic reduction of the ulna determines the reduction of the radial head spontaneously in 93% of the cases. In the other 7%, open reduction typically needs to be performed, finding the interposed annular ligament. If a close reduction of radial head dislocation is not possible after correct anatomic reduction and fixation of ulna fracture, an open reduction to remove soft tissue interposition is required.

Moreover, it was observed that when the reduction was not anatomic or not stable, the results were unsatisfactory. In our series, we observed appropriate treatment of the ulnar fracture. Regarding dislocation of the radial head, anatomic reduction of the dislocation of the radial head and immobilization during three weeks of pronosupination immobilization is advised.

In radial fractures Mason type I, the treatment should be orthopedic. In type II radial head fractures, anatomic reduction and stable fixation with early mobilization should be done.

According to previous research, in type III fractures, the excision, when indicated, should be delayed. If it is done at an early stage, a prosthetic limb that maintains the radial length should be used to avoid disruption of the radio-ulnar distal joint. In both cases, mobility should begin at an early stage.^20^

Our study has strengths and weaknesses. Among the strengths is the large population of adult patients having this uncommon injury followed-up for a long period of time, with a minimum clinical and radiological follow-up of 18 months. Our study has the inherent limitations of a retrospective series. Moreover, we were not able to fully analyze the characteristics of Bado type III and IV injuries, since, as expected from their low frequency and the adult population included, our series reported only four cases of such lesions.

## CONCLUSIONS

We have reported one of the largest series of patients with Monteggia lesion. It is a surgical urgency in which anatomic reduction and stabilization with the correct implants are essential for good outcomes. The risk factors for poor prognosis such as associated fractures (coronoid, radial head), nerve injury and open fractures should be promptly identified and considered, because they increase the risk of complications and need for surgical revision.
